# Valorization and Functional Enhancement of Mature Assam Tea Leaves Through Indigenous Filamentous Fungi-Based Fermentation for Functional Drink Development

**DOI:** 10.3390/foods15091562

**Published:** 2026-05-01

**Authors:** Kridsada Unban, Punnita Pamueangmun, Nang Nwet Noon Kham, Pratthana Kodchasee, Apinun Kanpiengjai, Chalermpong Saenjum, Kalidas Shetty, Chartchai Khanongnuch

**Affiliations:** 1 Division of Food Science and Technology, School of Agro-Industry, Faculty of Agro-Industry, Chiang Mai University, Chiang Mai 50100, Thailand; 2 Research Center for Multidisciplinary Approaches to Miang, Multidisciplinary Research Institute (MDRI), Chiang Mai University, Chiang Mai 50200, Thailand; chalermpong.s@cmu.ac.th; 3 Division of Biotechnology, School of Agro-Industry, Faculty of Agro-Industry, Chiang Mai University, Chiang Mai 50100, Thailand; punnita_pamu@cmu.ac.th (P.P.); nwenoonkham@gmail.com (N.N.N.K.); pratthana_kod@cmu.ac.th (P.K.); 4 Division of Biochemistry and Biochemical Innovation, Department of Chemistry, Faculty of Science, Chiang Mai University, Chiang Mai 50200, Thailand; apinun.k@cmu.ac.th; 5 Department of Pharmaceutical Sciences, Faculty of Pharmacy, Chiang Mai University, Chiang Mai 50200, Thailand; 6 Research Center for Innovation in Analytical Science and Technology for Biodiversity-Based Economic and Society (I-ANALY-S-T_B.BES-CMU), Multidisciplinary Research Institute (MDRI), Chiang Mai University, Chiang Mai 50200, Thailand; 7 Department of Microbiological Sciences, North Dakota State University, Fargo, ND 58102, USA; kalidas.shetty@ndsu.edu; 8 Center of Excellence in Agricultural Innovation for Graduate Entrepreneur, Maejo University, Chiang Mai 50290, Thailand

**Keywords:** fermented tea leaves, filamentous fungi growth-based process, bioactive compound, mature tea leaves, functional drink

## Abstract

Miang, a traditional fermented tea produced from *Camellia sinensis* var. *assamica*, is of notable cultural and socio-economic relevance in Northern Thailand. Traditionally, the non-filamentous fungi-based process (NFP) in western Lanna uses only young tea leaves, resulting in substantial amounts of mature leaves being discarded as agricultural waste. This study aimed to utilize the mature tea leaves by adapting the filamentous fungi growth-based process (FFP) of eastern Lanna using selected tannin-tolerant microorganisms, including *Aspergillus niger* MLF3, *Cyberlindera rhodanensis* P3, and *Lactiplantibacillus pentosus* A14-6. Study on fermentation dynamics and bioactive compound formation based on a 2-step fermentation process: 3-day solid-state fermentation with *A. niger* MLF3, followed by 7-day submerged fermentation by co-culture of *C. rhodaninsis* P3, and *L. pentosus* A14-6 in 500 mL sterile distilled water at 30 °C. Increased activities of polysaccharide-degrading enzymes and organic acids were clearly observed during solid-state fermentation, while the significant changes in polyphenol, antioxidant, and reducing sugar content in cell-free supernatant (CFS) were found after submerged fermentation. The obtained CFS shows inhibitory effects of 90 ± 2.5% and 95 ± 1.8% on α-glucosidase and α-amylase, respectively. Analysis of CFS by E-tongue and E-nose clearly indicated the influence of microbial mixture on the taste and aroma of the fermented products. These results demonstrate not only a high-yielding strategy for the effective biotransformation of mature tea leaves into functional drink products but also significant implications for reducing agricultural waste.

## 1. Introduction

Fermentation is one of the oldest methods of food preservation. It is also a low-cost energy conservation system that is essential to ensuring food safety and quality. Many biochemical changes occur during fermentation and may affect the nutrient composition and, consequently, the properties of the final product, such as bioactivity and digestibility [1]. Recently, this bioprocess has been applied for the production and extraction of bioactive compounds from plants in the food and beverage industries. Among fermented foods derived from plant materials, indigenous fermented tea has been well recognized as one of the fermented food products that contain a variety of bioactive compounds, providing a beneficial contribution to human health [2,3]. During microbial fermentation of tea, the content of catechins and flavonoids generally decreased, mainly due to oxidation or degradation of these compounds into gallic acid [4]. The bioactivities of unfermented tea and microbially fermented tea differ substantially. After fermentation, the antibacterial and antioxidant capacities of fermented tea are enhanced. Microbial fermentation has been reported to enhance antibacterial activity and antioxidant capacity [5,6,7]. In addition, microbially fermented teas have been associated with anti-inflammatory effects and potential weight-loss benefits [2]. Compared with unfermented tea, microbial fermentation can improve tea taste by promoting biochemical transformations. During fermentation, tea proteins break down into free amino acids, which enhance flavor. At the same time, catechins are degraded, contributing to a reduction in bitterness in the tea beverage [4,8,9,10]. In several well-known microbially post-fermented teas, filamentous fungi have been identified as key microorganisms driving these transformations. For example, a number of filamentous fungi were identified and shown to be important in the post-fermentation of Chinese dark teas, including Pu-erh, Fuzhuan, and Qingzhuan teas [11,12]. Moreover, unlike Chinese dark tea, some traditional fermented teas involve both aerobic and anaerobic fermentation and contain filamentous fungi, yeasts, and lactic acid bacteria. Depending on the process used, it has been identified across diverse regions under different names, such as Suancha or Yancha in China, Laphet in Myanmar, Goishi-cha and Awaban-cha in Japan, and Miang in Thailand [13,14,15].

Miang is a naturally fermented tea made from the leaves of *Camellia sinensis* var. *assamica* and has had prominent ethnic, cultural, and socio-economic relevance for many centuries among the Lanna people of northern Thailand and the surrounding region. Miang, fermented tea leaves without added substances, has also been traditionally consumed in northern Thailand for decades. It has been recognized as a phenolic metabolites-rich fermented food and has recently gained interest in the development of functional food ingredients and cosmetic products. The fermentation process of Miang involves a filamentous fungi-based process (FFP), mostly used in Phrae and Nan provinces (eastern Lanna), in which mature tea leaves serve as the substrate. In contrast, the non-filamentous fungi-based process (NFP) using young tea leaves as raw material is commonly used in the provinces of Chiang Mai, Chiang Rai, Lampang, and Mae Hong Son (western Lanna) [3]. In addition, because only young tea leaves are used for NFP in western Lanna, the mature tea leaves must be removed from tea plants during the dry season (January–March) each year to activate the spring of the new leaves, resulting in a large amount of unused mature tea leaves that are considered agricultural waste. Due to seasonal variation in metabolites, summer-harvest teas are considered more bitter and astringent than spring tea and are therefore less preferred for tea production [16,17].

Previous studies have contributed to the overall understanding of Miang microbial communities, and results suggest that lactic acid bacteria (LAB) and yeast play the most important roles in Miang fermentation, leading to biotransformation during fermentation and contributing to the special properties of Miang products [18]. In FFP Miang, the predominant microbial groups include filamentous fungi, yeasts, LAB, and *Bacillus* spp. Enzymes associated with plant cell-wall degradation (pectinases, cellulases, and β-mannanase) were mainly detected during the initial (aerobic) stage of fermentation. In addition, key organic acids identified in FFP Miang include acetic, gallic, glucuronic, lactic, oxalic, and succinic acids, which represent major chemical constituents of the product [19]. According to our previous studies, three groups of microbes are importantly involved in Miang fermentation process particularly specific species of *Aspergillus niger*, *Cyberlindnera rhodanensis*, and *Laciplantibacillus pentosus* [18,19]. These Miang-associated microbes, *A. niger* MLF3, *C. rhodanensis* P3 and *L. pentosus* A14-6, have been isolated for further study.

Based on knowledge of the Miang fermentation process and the microorganisms involved during the fermentation period of FFP Miang, filamentous fungi are the key microbes that can convert polysaccharide compounds from tea leaves into fermentable sugars by producing polysaccharide-degrading enzymes. These fermentable sugars can be further fermented by other microbes, such as lactic acid bacteria, *Bacillus*, or yeast, to produce health-beneficial metabolites, including organic acids, short-chain fatty acids, and catechin derivatives, which confer health benefits to humans. Recently, tannin-tolerant microorganisms, including filamentous fungi, lactic acid bacteria, and yeast, have been successfully isolated from Miang [20,21]. These microbes have attracted interest in their ability to survive and grow in tannin-rich substrates such as tea leaves and to produce beneficial metabolites and biotransformed products in tea-based substrates, including organic acid and derivatized phenolic compounds. Therefore, this research aimed to investigate the use of these specific microbes for the valorization of mature tea leaves, a by-product of Miang production, through the FFP to produce a functional drink with potential health benefits. The role of the selected filamentous fungi, in combination with the selected yeast and lactic acid bacteria, in altering the physical and chemical properties during fermentation was described. Moreover, the functional potential, including antioxidant activity, α-glucosidase and α-amylase inhibition activities, and preliminary safety assessed by cell cytotoxicity, was also demonstrated.

## 2. Materials and Methods

### 2.1. Microorganisms and Culture Conditions

*Aspergillus niger* MLF3, *Cyberlindnera rhodanensis* P3, and *Lactiplantibacillus pentosus* A14-6 were isolated from fermented Miang samples and maintained as stock cultures in 25% (*v*/*v*) glycerol at −80 °C [19,20,21]. The strains of *A. niger* MLF3, *C. rhodanensis* P3, and *L. pentosus* A14-6 were reactivated by streaking onto potato dextrose agar (PDA) (HiMedia, Mumbai, India), yeast malt (YM) agar (HiMedia, Mumbai, India), and de Man, Rogosa, and Sharpe (MRS) agar (HiMedia, Mumbai, India), respectively, for further use. To prepare the seed inoculum, the mature spores of *A. niger* MLF3 formed on PDA incubated at 30 °C for 5 days were collected, diluted with sterile 0.85% (*w*/*v*) NaCl to achieve 10^4^ spores/mL, and used as the seed inoculum. The cells of *L. pentosus* A14-6 cultured in MRS broth (HiMedia, Mumbai, India) at 37 °C for 24 h were harvested by centrifugation at 8000× *g* for 15 min and washed twice with 0.85% (*w*/*v*) NaCl solution. The cell pellets were then resuspended in 0.85% (*w*/*v*) NaCl to obtain a concentration of 10^8^ cells/mL. For *C. rhodanensis* P3 seed preparation, a single colony of yeast was inoculated in YM broth (HiMedia, Mumbai, India) and incubated at 30 °C on a 150-rpm rotary shaker for 48 h. Then, the cells were harvested by centrifugation at 8000× *g* for 10 min at 4 °C, washed twice with 0.85% (*w*/*v*) NaCl solution, and resuspended in the same solution to obtain a concentration of 10^7^ cells/mL.

### 2.2. Solid-State Fermentation of Mature Tea Leaves by Aspergillus niger MLF3

Mature tea leaves were subjected to solid-state fermentation using a filamentous fungi growth-based process (FFP), following the traditional method described by Kodchasee et al. [19] with some modifications. Briefly, fresh mature Assam tea leaves (Camellia sinensis var. assamica) collected (during March–April 2024) from the tea production area in Mae Tang, Chiang Mai Province, Thailand, were washed, and 20 g portions of leaves were tied into bundles with a bamboo strip and steamed for three hours. Then, 500 g of tea leaves were placed in a 1 L container with the lid closed, and the container was sterilized at 121 °C for 20 min. Then, 5% (*v*/*v*) of a spore suspension of A. niger MLF3 was sprayed onto the tea leaf sample, which was incubated for 10 days in a sealed 1 L container at 30 °C in a temperature-controlled incubator with 50–55% relative humidity (RH). During fermentation, tea leaves were aseptically removed from the container each day in 20 g bundles to assess microbial enumeration, pH, polysaccharide-degrading enzyme activity, polyphenol content, antioxidant activity, and reducing sugar content. The analysis was performed according to the method described in the previous report with slight modifications [19]. Briefly, 20 g of the fermented tea sample was mixed with 200 mL of sterile water and homogenized for 10 min using a Masticator homogenizer (IUL Instruments, Barcelona, Spain). The homogenized sample was used for microbial enumeration and pH measurement. In addition, an aliquot of the homogenate was centrifuged at 8000× *g* and 4 °C for 10 min. The resulting supernatant was collected and used to determine polysaccharide-degrading enzyme activity, polyphenol content, antioxidant activity, and reducing sugar content.

### 2.3. Mature Tea Leaves Co-Fermentation

The 500 g of steamed mature tea leaves were first subjected to solid-state fermentation with *Aspergillus niger* MLF3 at 30 °C for 3 days in a 1 L container. The fermented leaves were then inoculated with 1% (*v*/*v*) seed cultures of lactic acid bacteria and/or yeast (as specified by each treatment), and 500 mL of sterilized water was added. Seven treatments were tested in each experimental set: L, *L. pentosus* A14-6; Y, *C. rhodanensis* P3; F, *A. niger* MLF3; FY, co-inoculation with *A. niger* MLF3 and *C. rhodanensis* P3; FL, co-inoculation with *A. niger* MLF3 and *L. pentosus* A14-6; FLY, co-inoculation with all three strains; and C, no starter culture (control). Then, all treatments were incubated at 30 °C for 7 days. Each day during this period, culture broth was collected for microbial enumeration, polysaccharide-degrading enzyme activity, pH, color, organic acid content, antioxidant activity, and α-glucosidase and α-amylase inhibition activities.

### 2.4. Enumeration of Microorganisms, pH, and Color Changes

A volume of 1 mL of fermentation broth was serially diluted in sterile saline (0.85%). A volume of 10 μL of 10^−5^ to 10^−7^ dilutions was then dropped on MRS agar with 15 ppm bromocresol purple and YM agar with 0.1 g/L chloramphenicol for counting the viable cell number of LAB and yeast, respectively. The agar plates were incubated at 30 °C for 3 days, and the number of microbes was calculated as colony-forming units (CFU)/mL of sample and expressed in log CFU/mL. The pH of the supernatant was measured with a pH meter (Ohaus 2100, Ohaus Co., Parsippany, NJ, USA). The liquor color of the sample was measured using a HunterLab ColorQuest XE (Hunter Associates Laboratory, Reston, VA, USA). The values were measured in terms of lightness (L*) and color (+a*: redness, −a*: greenness, +b*: yellowness, −b*: blueness).

### 2.5. Determination of Polysaccharide-Degrading Enzymes

The fermentation broth was centrifuged at 8000× *g* at 4 °C for 10 min, and the supernatant was used for enzyme activities of cellulase, (mannanase, xylanase, amylase, and pectinase) using the 3,5-dinitrosalicylic acid (DNS) method. The sample volume of 0.125 mL were mixed with 0.25 mL of 0.5% (*w*/*v*) of substrate carboxymethyl cellulose (CMC) (Nacalai Tesque, Inc., Tokyo, Japan), locust bean gum (Sigmar-Aldrich, St. Louis, MO, USA), beech wood xylan (Megazyme, Wicklow, Ireland), soluble starch (KemAus, Cherrybrook, Australia) and pectin (Wako Pure Chemical, Tokyo, Japan), respectively, for all five enzyme assays. The reactions were carried out at 37 °C for 10 min, and 0.25 mL of DNS solution (Sigma-Aldrich, St. Louis, MO, USA) was added, followed by boiling for 5 min. The absorbance was measured at 540 nm using a UV-Vis spectrophotometer. Enzyme activity unit (U) was expressed as the amount of enzyme able to liberate 1 µmole of reducing sugar from the substrate per minute under the described conditions [20].

### 2.6. Determination of Organic Acids

The fermentation broth was analyzed for organic acids using high-performance liquid chromatography (HPLC). The sample was injected into the HPLC system (Agilent 1000 series, Agilent Technologies Inc., Palo Alto, CA, USA). The conditions were as follows: Rezex ROA organic acid H^+^ (8%) column (150 × 7.8 mm, Phenomenex, Torrance, CA, USA); mobile phase 5 mM H_2_SO_4_ and 2% acetonitrile were used with an isocratic elution of 50:50 (*v*/*v*) with a flow rate of 0.5 mL/min at temperature 40 °C and UV detector at 210 and 250 nm. The concentrations of organic acids in culture broth were determined by comparing their retention times (RTs) with those of standards (acetic, citric, gallic, glucuronic, lactic, malic, oxalic, succinic, and tartaric acids). The chromatograms of the standard mixture and fermentation broth sample are provided in Supplementary Figure S1.

### 2.7. Determination of Total Polyphenol Content

The total polyphenol content (TP) in the samples was determined using the Folin–Ciocalteu method [22], with some modifications. Briefly, 200 µL of 2 M Folin–Ciocalteu reagent was added to 200 µL of the sample, followed by 1.8 mL of deionized water, which was vortexed. Then, 400 µL of 10% (*w*/*v*) sodium carbonate was added, and the final volume was adjusted to 4 mL with deionized water. After 1 h of rest in the dark at room temperature, the absorbance of blue coloration was measured at 725 nm using a UV-Vis spectrophotometer. The total polyphenol content was calculated based on a gallic acid calibration curve and expressed as milligrams of gallic acid equivalents (GAE) per mL of sample.

### 2.8. Determination of Total Flavonoid Content

Total flavonoid (TF) in the sample was quantified using the aluminum chloride colorimetric assay with quercetin as the standard [22]. Briefly, 100 µL of 10% (*w*/*v*) aluminum nitrate was added to 500 µL of the sample, followed by 100 µL of 1 M potassium acetate and 3.3 mL of 80% (*v*/*v*) methanol. The mixture was then vortex-mixed thoroughly. Samples were incubated in the dark at room temperature for 40 min, and absorbance was measured at 415 nm using a UV-Vis spectrophotometer. The total flavonoid content was calculated based on the calibration curve of quercetin and expressed as milligrams of quercetin equivalents (QE) per mL of sample.

### 2.9. Determination of Total Tannin Content

Total tannin content (TT) was quantified using a modified Folin–Ciocalteu assay, with polyvinylpolypyrrolidone (PVPP) applied to selectively remove tannins and thereby distinguish them from non-tannin phenolic compounds. Briefly, 1 mL of the sample was mixed with 1 mL of 10% (*w*/*v*) PVPP, vortexed, and kept at 4 °C for 15 min. The reaction mixture was then centrifuged at 3000× *g* for 10 min, and the supernatant was collected. The remaining total phenol content of PVPP precipitated supernatant was measured with the Folin–Ciocalteu reagent, and TT was estimated by using the following formula: TT = TP-PVPP precipitation. The results were expressed as milligrams of tannic acid equivalents (TAE) per mL of sample.

### 2.10. Determination of Antioxidant Activity

#### 2.10.1. DPPH Radical Scavenging Activity Assay

The antioxidant activity of the sample was assessed using the 1,1-diphenyl-2-picrylhydrazyl (DPPH) free radical scavenging assay, as described by Abdullahi et al. [22], with slight modifications. Briefly, 1 mL of the diluted sample was mixed with 4 mL of 0.15 mM DPPH solution. After 30 min of incubation in the dark at room temperature, the absorbance was then measured at 517 nm. The DPPH scavenging capacity was expressed as Trolox equivalents per mL of sample.

#### 2.10.2. ABTS Radical Scavenging Activity Assay

The ABTS radical scavenging activity was determined according to Abdullahi et al. [22] with slight modifications. Briefly, the stock solution was prepared by mixing 7 mM of 2,2-azino-bis-3-ethylbenzothiazoline-6-sulphonic acid (ABTS) solution (in DI water) with 2.45 mM of potassium persulfate solution at a 1:0.5 (*v*/*v*) ratio, followed by incubation for 12 h in the dark at room temperature. The stock solution was diluted with DI water to obtain an absorbance of 0.700 ± 0.020 at 734 nm, which was used as the working solution. The diluted sample (20 µL) was mixed with 700 µL of the prepared working solution. The mixture was incubated in the dark for 10 min before measuring the absorbance at 734 nm with a microplate reader. The ABTS scavenging capacity was calculated using a Trolox calibration curve and expressed as Trolox equivalents (TE) per mL of sample.

### 2.11. Determination of α-Glucosidase Inhibitory Activity

The α-glucosidase inhibitory activities were determined using the method described by Yang and Kong. [23]. The diluted sample (100 µL) was mixed with 100 µL of α-glucosidase enzyme solution (1.0 U/mL prepared in 0.5 M phosphate buffer, pH 6.5) and 100 µL of substrate solution (2 mM *p*-nitrophenyl-α-D-glucopyranoside in 0.5 M phosphate buffer, pH 6.5), followed by incubation for 10 min at 37 °C. The reaction was stopped by adding 500 µL of 0.1 M Na_2_CO_3_. The absorbance of the reaction mixture was measured at 405 nm by a spectrophotometer. Distilled water mixed with the enzyme and substrate was used as a control. The relative α-glucosidase inhibitory activity (%) was calculated by the following equation:

α-glucosidase inhibition (%) = (A_control_ − A_sample_) × 100/A_control_

### 2.12. Determination of α-Amylase Inhibitory Activity

The α-amylase inhibition was carried out according to the method described by Leangnim et al. [24], with slight modifications. Briefly, the volume of 100 µL of diluted sample was mixed with 100 µL of α-amylase enzyme solution (100 U/mL prepared in 0.5 M phosphate buffer pH 6.5) and 100 µL of substrate solution (15 g/L of soluble starch in 0.5 M phosphate buffer pH 6.5), followed by incubation for 10 min at 37 °C. To terminate the reaction, 300 µL of 1 M HCl was added, followed by 2.4 mL of iodine solution. The absorbance was measured at 620 nm by a spectrophotometer. The results were expressed as percent α-amylase inhibition and calculated using the following equation:

α-amylase inhibition (%) = (A_control_ − A_sample_) × 100/A_control_

### 2.13. Measurement of Cell Viability

Human colon carcinoma cell line (Caco-2) and Human colon adenocarcinoma cell line (HT-29) obtained from the American Type Culture Collection (ATCC, Manassas, VA, USA) were used to carry out the cell cytotoxicity test. The cell viability was tested using the PrestoBlue^TM^ cell viability reagent (Life Technologies Corporation, Eugene, OR, USA). The volume of 100 μL cells (5 × 10^3^ cells/well) was seeded into each well of a 96-well plate and incubated at 37 °C for 24 h with 5% CO_2_. Then, the culture medium was replaced with serial dilution of samples (25–200 μg/mL) and incubated at 37 °C for 72 h. Then the culture supernatant was removed and incubated with 100 μL/well of the PrestoBlue solution at 37 °C for 2 h. The absorbance was measured at 560 and 595 nm using a microplate reader (SpectraMax M3, San Jose, CA, USA), and cell viability was calculated [25].

### 2.14. E-Tongue Analysis for the Taste

The sample was analyzed for taste using the E-tongue ASTREE (Alpha M.O.S., Toulouse, France). The sensor array consists of seven sensors, including AHS, ANS, SCS, CTS, and NMS, which are sensitive to sour, sweet, bitter, salty, and umami tastes, respectively, while PKS and CPS capture the overall taste profile [26]. The testing protocol was performed as follows: 30 s for cleaning, 120 s for testing, and 30 s for cleaning. The stable signal (between 110 and 120 s) was selected as the output value. Each sample was repeated three times.

### 2.15. E-Nose Analysis for the Volatiles

The volatile characteristics of the sample were analyzed using an E-nose Heracles NEO (Alpha M.O.S., Toulouse, France) equipped with an automatic headspace sampler. MXT-5 and MXT-1701 capillary columns separated the volatile organic compounds (VOCs). The VOCs were detected with two flame ionization detectors (FIDs). E-nose analysis was performed according to the method created by the following parameters of the PAL-RSI Autosampler and the Heracles GC analyzer. Briefly, 2 mL of sample in a 20 mL headspace vial capped with a magnetic PTFE/silicone was incubated at 60 °C for 20 min with a 500 rpm agitation rate. After incubation, 5 mL of the headspace gas was injected into the Heracles analyzer, with a 90 s flushing time between injections. Helium was used as a carrier gas with a flow rate of 30 mL/min. The VOCs were trapped at 50 °C and the initial oven was maintained at 50 °C for 5 s, ramped up to 80 °C at a rate of 1 °C/s, then further increased to 250 °C at a rate of 3 °C/s, holding for 21 s. The operational temperature for two FIDs was set at 260 °C. The chromatograms were converted into individual variables in AlphaSoft software version 17.0 (Alpha M.O.S., Toulouse, France) based on the areas and respective Kovats indices of the identified peaks. The compounds were analyzed using the AroChemBase database version 2021 (Alpha MOS Corporation, Toulouse, France). Three replicates were conducted on each sample.

### 2.16. Statistical Analysis

All experiments were performed in triplicate and represented as the mean ± standard deviation. Statistical analyses were conducted in IBM SPSS Statistics 23.0 (SPSS Inc., Chicago, IL, USA) using Tukey’s multiple-range test at a significance level of *p* < 0.05. GraphPad Prism version 9.4.0 (GraphPad Software Inc.; San Diego, CA, USA) was used to visualize the graphs.

## 3. Results and Discussion

### 3.1. Physicochemical Properties and Biological Properties of Mature Tea Leaves During Solid-State Fermentation by Aspergillus niger MLF3

**Figure 1:**
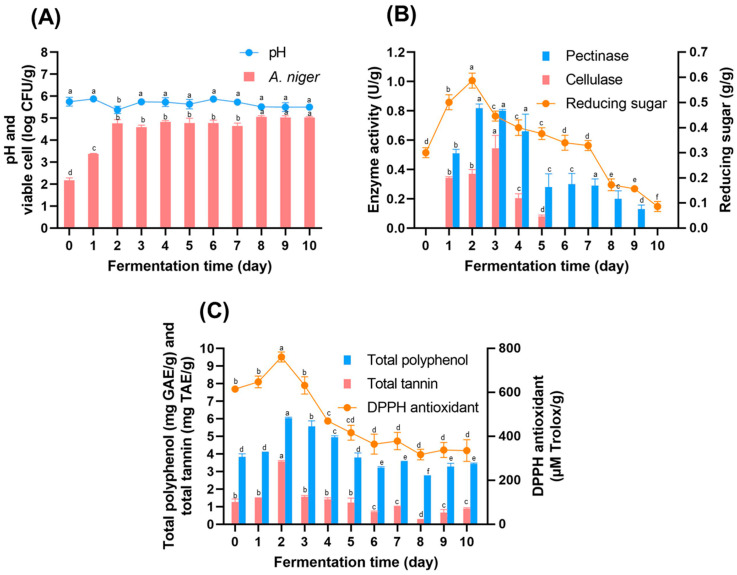
The pH and viable cell count (**A**), polysaccharide-degrading enzyme and reducing sugar content (**B**), and total polyphenol and total tannin content (**C**) of mature tea leaves fermented with *A. niger* MLF3 at 30 °C for 10 days. Different lowercase letters indicate significant differences among fermentation times within the same parameter (*p* < 0.05).

During aerobic fermentation, *A. niger* MLF3 was observed on the tea leaves surface on the second day and remained throughout the fermentation period. At the same time, the fermented broth showed spore counts ranging from 4 to 5 logCFU/g (Figure 1A). The tea fermentation broth showed the highest levels of pectinase and cellulase after 3-day fermentation, coinciding with the increase in reducing sugars during this period (Figure 1B). In addition, the bioactive compound and antioxidants showed higher concentrations after 3 days of fermentation (Figure 1C). The color of the mature tea leaves was yellow-brown and darker with longer fermentation, presumably due to the mature spores of *A. niger* MLF3. The dark colors of mature tea leaves may not be suitable for product development. Therefore, fermented tea leaves were selected after 3-day fermentation for the next experiment because high levels of enzymes and reducing sugars, along with high amounts of bioactive compounds, were detected, and a non-dark color was observed. This study simulated an experiment similar to the FFP Miang fermentation process, using mature tea leaves as the raw material. *A. niger* MLF3, which plays a crucial role in producing polysaccharide-degrading enzymes, especially pectinase, was used. These enzymes make the soft texture of FFP Miang. Additionally, these enzymes break down the tea leaf structure, releasing sugars that serve as a carbon source for other microorganisms during fermentation [19,27]. This mechanism is particularly relevant for producing fermented health beverages, as it can support microbial growth and fermentation without the need to add external sugars.

### 3.2. Microbial, pH, and Color Changes During Co-Fermentation

**Figure 2:**
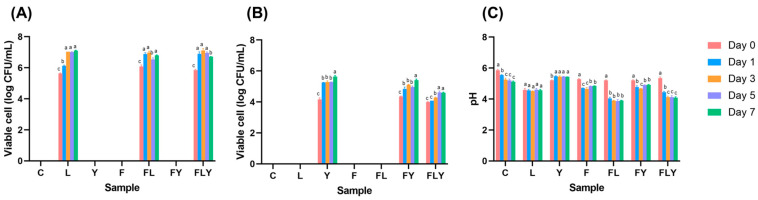
The viable cell counts of lactic acid bacteria (**A**), yeast (**B**), and pH (**C**) change during co-fermentation at 30 °C for 7 days. Different lowercase letters indicate significant differences among fermentation times within the same treatment (*p* < 0.05).

During anaerobic fermentation, the population of *C. rhodanensis* P3 yeast and *L. pentosus* A14-6 increased rapidly from day 0 to day 3 (Figure 2). *C. rhodanensis* P3 increased from 4 to 5 logCFU/mL on the first day of fermentation (*p* < 0.05) and remained constant for the remainder of the fermentation period (*p* > 0.05). In contrast, *L. pentosus* A14-6 increased from 5 to 7 logCFU/mL. In the case of the fermented broth with *A. niger* MLF3, no growth was observed under these conditions (Figure 2). However, when comparing the type and number of microorganisms found during single fermentation and co-fermentation with *A. niger* MLF3, it was observed that *L. pentosus* A14-6 grew more quickly during fermentation days 0 to 2 than during single fermentation. It is expected that *A. niger* MLF3 can produce enzymes that digest polysaccharides, breaking down the tea leaf structure into sugar, which other microorganisms can then utilize for growth. Additionally, *L. pentosus* A14-6 was able to convert more of these sugars into organic acids, as evidenced by the rapid decrease in pH from 6 to 4 within the first day of co-fermentation with *A. niger* MLF3. In contrast, single-fermentation with *A. niger* MLF3, *C. rhodanensis* P3, and *L. pentosus* A14-6 maintained a constant pH of approximately 5, 6, and 5.5, respectively (Figure 2C). These observations of microbial growth agree with previous reports that all the used microbes grew in high-tannin substances and enhanced the production of organic acids through microbial metabolism and enzymatic conversion of tea leaves [19,20,21].

**Figure 3:**
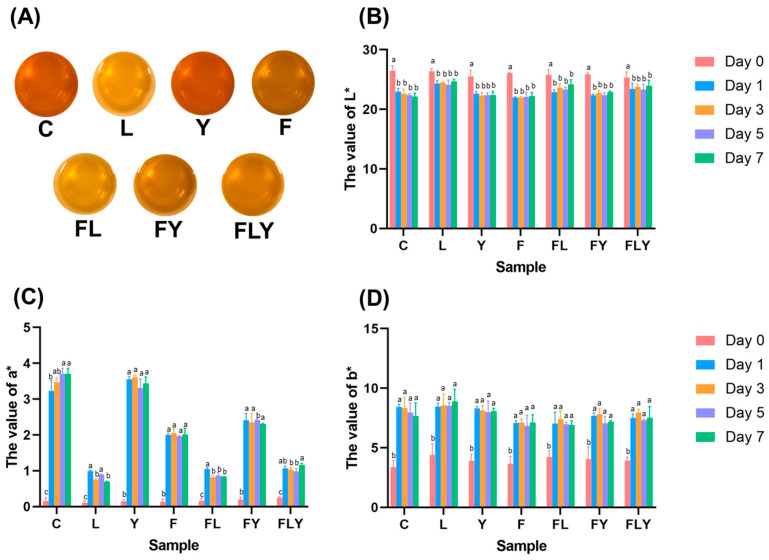
The appearance of the co-fermented mature tea leaves sample at day 7 (**A**) and the color value of L* (**B**), a* (**C**), and b* (**D**). Different lowercase letters indicate significant differences among fermentation times within the same treatment (*p* < 0.05).

The color of the co-fermented mature tea leaves sample, and the color value, is presented in Figure 3. Fermentation with starter cultures significantly altered the infusion color (L*, a*, and b*) during incubation at 30 °C for 7 days. Compared with day 0, all treatments showed reduced L* values after fermentation, indicating progressive darkening of the tea liquor. In parallel, a* and b* increased markedly from day 0 to day 1 and remained relatively stable through days 3–7, which suggested rapid formation of yellow–red chromophores early in fermentation. These changes may be attributed to microbial biotransformation of tea polyphenols and enhanced oxidative polymerization reactions. This could be promoted by extracellular enzymes, particularly in *A. niger* MLF3 treatments. Moreover, LAB treatment would acidify the culture broth, providing synergistic effects on color development.

### 3.3. Polysaccharide-Degrading Enzyme Activity During Co-Fermentation

**Figure 4:**
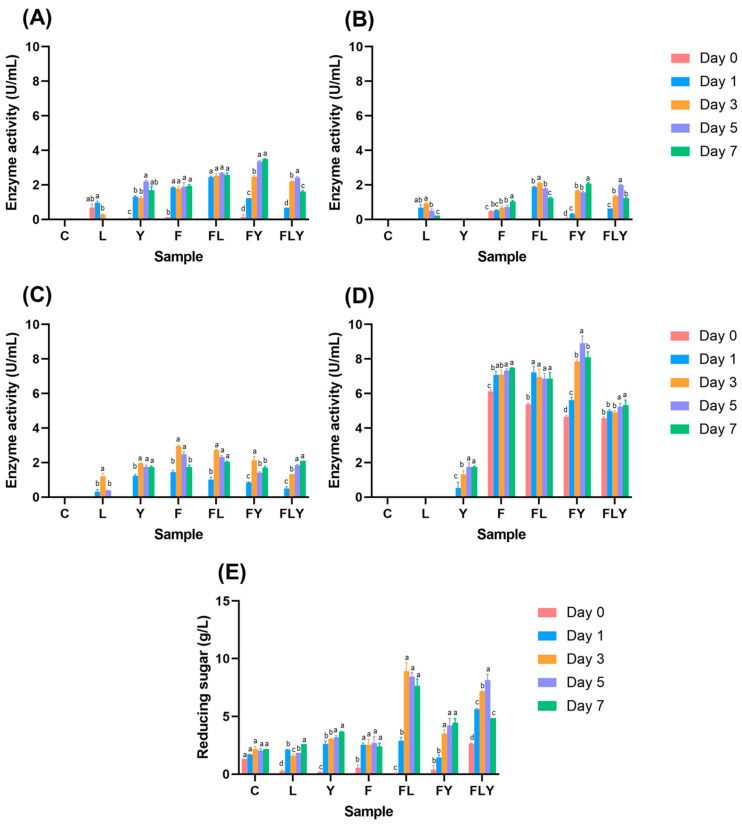
Polysaccharide-degrading enzymes of mannanase (**A**), cellulase (**B**), xylanase (**C**), pectinase (**D**) activity, and reducing sugar content (**E**) during co-fermentation at 30 °C for 7 days. Different lowercase letters indicate significant differences among fermentation times within the same treatment (*p* < 0.05).

Determination of enzymatic activity in fermented tea broth revealed that *A. niger* MLF3 produced pectinase, cellulase, xylanase, and mannanase, with pectinase as the predominant enzyme (Figure 4). The pectinase activity was consistently high in the single-fermentation with *A. niger* MLF3, averaging 7.30 ± 0.55 U/mL from day 0 to day 7 (*p* < 0.05). In the co-fermentation of *A. niger* MLF3 with *C. rhodanensis* P3, the pectinase activity increased after 1 day of fermentation, from 4.71 ± 0.14 U/mL to 8.90 ± 0.72 U/mL. The co-fermentation of *A. niger* MLF3 and *L. pentosus* A14-6 showed a maximum pectinase activity of 8.75 ± 0.72 U/mL initially, but it decreased after 3 days of fermentation. This decrease might be attributed to the low pH, which could potentially inhibit enzyme activity. During co-fermentation with all microbes, pectinase activity was detected after 3 days, increasing significantly from 2.35 ± 0.43 U/mL to 6.42 ± 0.29 U/mL (*p* < 0.05). The differences in the co-fermentation results could be due to the relationships between the microorganisms observed in the single-fermentation experiments. The fermented broth with *C. rhodanensis* P3 showed pectinase activity after 3 days, averaging 1.39 ± 0.22 U/mL, while no pectinase activity was detected in the fermented broth with *L. pentosus* A14-6. The study also revealed that the enzymes produced by the microorganisms increased reducing sugar levels in the fermented Mature tea leaves broth compared to the control. The reducing sugars found in the control samples might be due to heating during the sterilization of the Miang leaves, which could have increased the sugar content, which then dissolved in the Mature tea leaves broth, resulting in a relatively constant value.

In contrast, the fermented broth showed both increases and decreases in reducing sugar levels. This suggests that microbial fermentation contributed to dynamic changes in the reducing sugar content of the fermentation broth, as microorganisms both consumed and released sugars through enzymatic activities and metabolic processes. Moreover, it has also been reported that the carbohydrate-degrading enzymes produced by filamentous fungi could convert conjugated phenolic compounds to free form, improving their capacity to function as good antioxidants, especially the enzymes produced by *Aspergillus* can improve their antioxidant activity in the food and beverage industry such as black rice bran [28], plum fruit [29], rice Koji [30] and Chinese dark tea [31]. However, the traditional and modern plant-based food fermentation industries frequently co-ferment filamentous fungi with other microorganisms to improve the quality of the co-fermented products. This process involves many factors, such as the production of organic acids, pH changes, enzyme secretion, and reactions with food substrates.

### 3.4. Bioactive Compounds, Antioxidant Activity, and Organic Profiles of Co-Fermented Mature Tea Leaves

**Figure 5:**
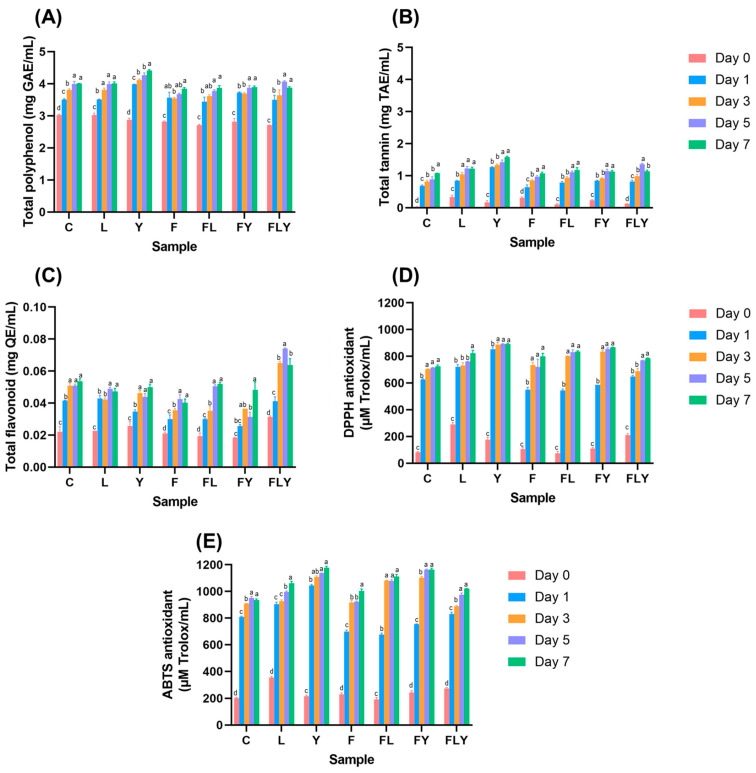
Total polyphenol (**A**), total tannin (**B**), and total flavonoid (**C**) contents and antioxidant activity determined by DPPH (**D**) and ABTS (**E**) during co-fermentation at 30 °C for 7 days. Different lowercase letters indicate significant differences among fermentation times within the same treatment (*p* < 0.05).

Determination of bioactive compounds in mature tea leaves broth revealed total polyphenol, total tannin, and total flavonoid in all experimental samples. The amount of substances ranged from 2.70 ± 0.03 to 4.41 ± 0.03 mg/mL, 0.10 ± 0.03 to 1.58 ± 0.03 mg/mL, and 0.02 ± 0.00 to 0.07 ± 0.01 mg/mL, respectively. The antioxidant activity with values in the range of 73.32 ± 2.60 to 893.90 ± 4.33 mM Trolox/mL (DPPH assay) and 202.55 ± 5.80 to 1176.66 ± 11.47 mM Trolox/mL (ABTS assay). The antioxidant activity assessed by the ABTS assay showed a trend consistent with the DPPH result, with the highest Trolox equivalent antioxidant capacity observed on day 7. The highest trend of bioactive compounds was the single fermentation with *C. rhodanensis* P3, followed by the co-fermentation of *A. niger* MLF3 and *C. rhodanensis* P3 (Figure 5). The analysis of organic acids in fermented mature tea leaves broth samples revealed the presence of oxalic acid, glucoronic acid, and gallic acid. Lactic acid was present in all the experimental sets except the control. Importantly, the gallic acid content increased significantly in *C. rhodanensis* P3 (Table 1), which may be related to the increased antioxidant activity observed in the fermented mature tea leaves broth with this strain. The polyphenol and flavonoid contents in tea are commonly recognized as the source of bioactivity. Mature tea leaves are particularly rich in catechins (such as C, EC, ECG, EGCG, GC), flavonols (myricetin, quercetin, kaempferol), and phenolic acids (caffeic, chlorogenic, coumaric, and sinapic acids), which are associated with positive health effects [32,33,34]. During fermentation, the concentrations of some polyphenols and flavonoids might initially decrease due to enzymatic oxidation and microbial utilization. Fermentation also influences levels of other bioactive compounds, such as tannins and proanthocyanidins, which may contribute to both the taste and the functional properties of the tea [22,33,35]. The antioxidant activity of tea is primarily attributed to its polyphenols and flavonoids. The previous report noted that antioxidant capacity decreased as fermentation progressed [22,33,34]. This reduction has been associated with the breakdown and transformation of catechins and other potent antioxidants. Nonetheless, the increase in certain phenolic acids, such as caffeic and sinapic acids, during extended fermentation suggests that a new mechanism in which antioxidants might form as others degrade [22,32].

### 3.5. α-Glucosidase and α-Amylase Inhibitory Activity

**Figure 6:**
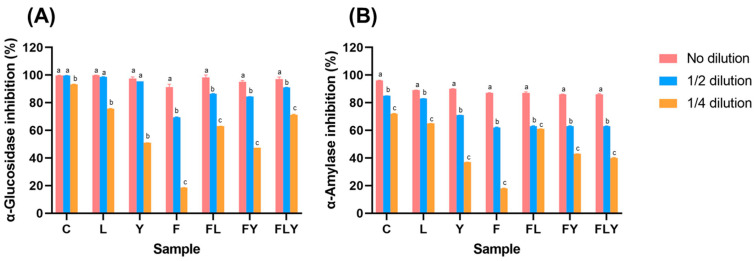
Inhibitory activity of co-fermented mature tea leaves against the enzyme activities of α-glucosidase (**A**) and α-amylase (**B**). Different lowercase letters indicate significant differences among fermentation times within the same treatment (*p* < 0.05).

Regarding the evaluation of the inhibitory effects on the enzymes α-glucosidase and α-amylase, which are related to the reduction in blood sugar levels in type 2 diabetic patients, the fermented mature tea leaves samples showed inhibition rates in the range of 20–90% and 14–95% (Figure 6), respectively, for the two enzymes. Interestingly, the control samples exhibited the highest inhibition rates for both enzymes. The control sample could be attributed to the presence of catechins in the mature tea leaves, which the microbial fermentation may not have significantly altered. Further investigation is warranted to elucidate this observation. Overall, the fermented, mature tea leaf broth demonstrated promising inhibitory effects on key enzymes involved in carbohydrate metabolism, suggesting its potential as a natural approach to managing type 2 diabetes. However, the higher inhibitory activity observed in the control samples warrants additional research to understand the underlying mechanisms and the specific bioactive compounds responsible for these beneficial effects. A compound tea fermented with *A. niger* showed increased levels of active compounds and enhanced inhibitory activities against α-glucosidase and α-amylase [36,37,38]. Tea polyphenols, including catechins and theaflavins, play a crucial role in inhibiting α-glucosidase and α-amylase. They interact with enzymes via hydrogen bonding and hydrophobic interactions, altering enzyme structure and reducing their activity [39]. The fermentation process enhances the concentration of bioactive compounds, which correlates with increased enzyme inhibition. Optimal fermentation conditions vary, but lower temperatures generally yield higher inhibitory activities [37,40,41].

### 3.6. Cytotoxicity of Co-Fermented Mature Tea Leaves on Caco-2 and HT-29 Cells

**Figure 7:**
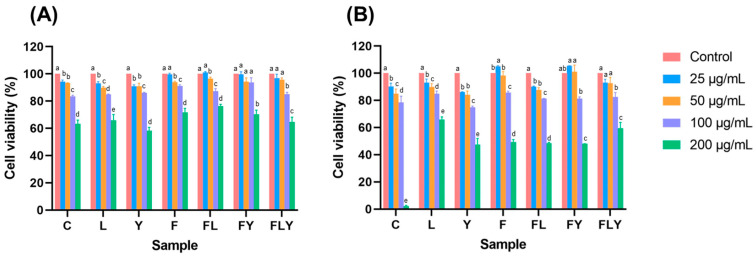
The cytotoxicity of Caco-2 cells (**A**) and HT-29 cells (**B**) after treatment with co-fermented mature tea leaves samples for 72 h. Different lowercase letters indicate significant differences among fermentation times within the same treatment (*p* < 0.05).

The cytotoxicity degree of the co-fermented mature tea leaves samples on Caco-2 and HT-29 cell proliferation was assessed after 72 h of treatment at various concentrations (Figure 7). The results showed that 25–100 µg/mL of fermented mature tea leaf samples (F, L, Y, FL, FY, and FLY) did not cause cytotoxicity in either cell line, with cell viability remaining >80% and comparable to that of the control (C). These findings suggest that fermented mature tea leaves have potential for further development as functional health products, with no apparent adverse effects on human cells under the conditions tested.

### 3.7. E-Tongue Analysis

**Figure 8:**
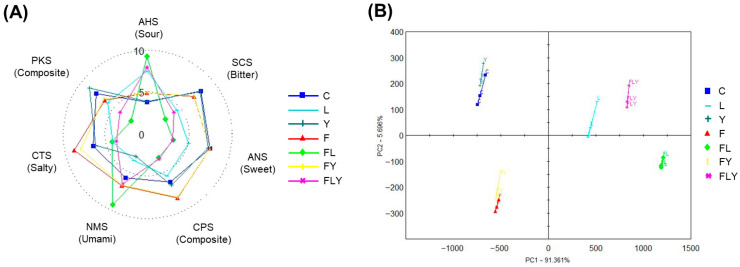
The radar chart of the E-tongue test (**A**) and principal component analysis (**B**) based on the E-tongue test result.

The results from E-tongue analysis demonstrated that microbial fermentation significantly altered the tea taste profile, as shown in Figure 8. The radar plot indexes for all tastes showed significant differences (Figure 8A). Among all taste indexes, the sourness of the fermented products FL, FLY, and L was higher than that of the others, which might be responsible for the presence of lactic acid bacteria. The strongest umami taste was observed in treatment FL, while treatments F, FY, and FLY also exhibited higher umami intensity than the control. Moreover, bitterness was most strongly reduced in treatment FL, followed by FLY, L, FY, and F, relative to the control. PCA further confirmed clear discrimination among all treatments (discrimination index 99), suggesting that each starter culture produced a reproducible and distinct taste signature. The PCA score plot shown in Figure 8B illustrates the classification of seven distinct sample varieties, with PC1 and PC2 accounting for 97.05% of the cumulative variance. Overall, these results indicate that microbial fermentation is a key driver of changes in the taste sensory profile. Previous studies on microbial transformation during tea-leaf fermentation have shown that fungal and bacterial activities can reduce astringency and lingering astringent aftertaste by decreasing catechin levels. These microorganisms can produce proteases, tannases, and other hydrolytic enzymes, which play important roles in modifying compounds responsible for the bitter and astringent taste of dark tea [42,43,44]. Some reports also indicated that starter cultures play a key role in shaping the flavor and sensory traits of fermented products [45,46]. Lee et al. [47] reported that *Lactobacillaceae* starters were involved in the concentration change in amino acid derivatives during kimchi fermentation. E-tongue results indicated increased sourness and decreased sweetness and bitterness across all samples, while umami notably increased only with a specific starter.

### 3.8. E-Nose Analysis

**Figure 9:**
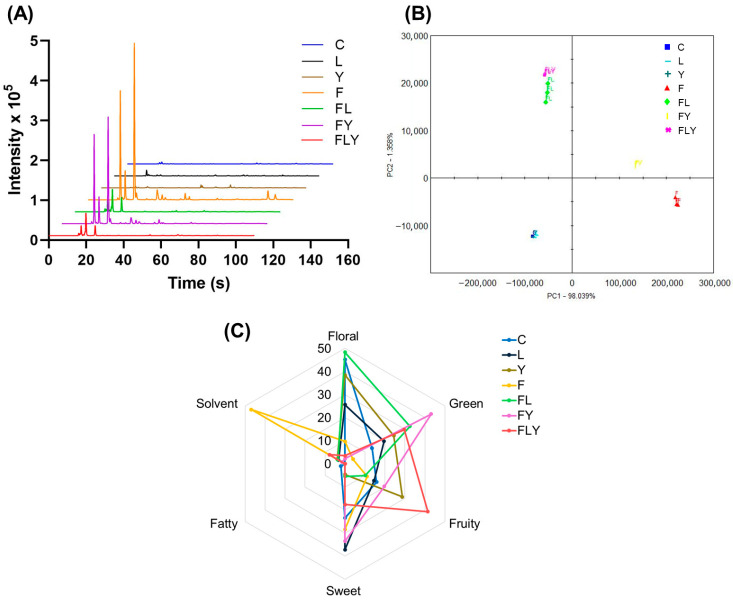
The gas chromatogram overlay diagram (**A**), principal component analysis based on E-nose results (**B**), and radar map of aroma profile (**C**) of E-nose detection results.

Since the overlay chromatograms obtained from the MXT-1701 column showed trends similar to those from the MXT-5 column, the MXT-5 chromatographic output was selected as the representative dataset, as shown in Figure 9A. Overall, the strongest signals clustered in the early retention time around 20–60 s. Changes in peak intensity and distinct peaks were observed between treatments, indicating alterations in volatile abundance and composition after fermentation. Further separation among treatment groups was then evaluated using principal component analysis (PCA). As shown in Figure 9B, the PCA score plot shows that the horizontal and vertical axes correspond to PC1 and PC2, respectively. PC1 accounts for 98.04% of the total variance, while PC2 explains 1.35%, indicating that the first two principal components capture nearly all of the information required to represent the overall sample characteristics. The discrimination index reaches 98, demonstrating that measurable differences exist among the VOC profiles of the sample groups and that the electronic nose method provides strong discriminatory capability. As observed in the plot, FL and FLY cluster closely together, suggesting minimal overall odor differences between these two groups, whereas F was clearly separated from the others along PC1, indicating a comparatively distinct VOCs composition. Additionally, chromatographic peaks were subjected to qualitative analysis using the AroChemBase database (Supplementary Table S1). The levels of key aroma compounds, such as ethyl acetate, isoamyl acetate, and linalool, increased compared with the control treatment. Moreover, the identified VOCs were also confirmed by MXT-1701 columns. Our results were consistent with previously reported findings that *C. rhodanensis* P2 produces the main VOC, ethyl acetate (fruity aroma), which can help improve product flavor [44]. Other *Cyberlindnera* spp. also have been reported to produce important VOCs [48,49].

In addition, the radar chart (Figure 9C) suggests distinct aroma profile trends among treatments. For example, F showed a markedly higher solvent-like profile, whereas FY exhibited the strongest green note. FLY displayed the highest fruity profile, while FL showed a prominent floral profile. Overall, fatty-related signals were comparatively low across most samples. These results suggest that co-fermentation helps improve the overall odor of fermented tea. Similar qualitative trends have been described for other microbially fermented teas, where fermentation is associated with a reduction in green notes and an enhancement of fruity or sweet notes due to the formation of new volatile metabolites [41,42,43]. *Aspergillus* species have been previously reported to play an important role in enzymatic hydrolysis, producing enzymes that break down the tea leaf structure for biotransformation into VOCs [50,51]. Moreover, it is associated with the oxidative polymerization of essential tea constituents, owing to their greater sensitivity, higher productivity, and lower side reactions [52,53]. Lactic acid bacteria and yeast were also associated with various enzymes, including polyphenol oxidases and other oxidases, in pickle tea aromas [52,54]. Yeasts, especially non-*Saccharomyces* yeasts, have been increasingly explored to enhance the formation of flavor- and odor-active compounds during fermentation [55,56,57]. Many species are of particular interest because they produce β-glucosidase, which can promote aroma development by hydrolyzing glycosidic bonds in monoterpene glycosides and other bound precursors, thereby releasing free aromatic compounds into fermented products such as wine and black tea [58,59,60]. The use of residual Miang fermentation broth as a by-product for developing health-oriented beverages using selected non-*Saccharomyces* yeasts has also been reported [44]. Based on the overall results, fungi appear to play an important role in aroma formation through their ability to degrade tea leaves. As reported previously, Miang tea leaf fermentation via the FFP identified fungi as key microorganisms responsible for polysaccharide breakdown, releasing monosaccharides that can be utilized by yeasts and bacteria and subsequently serve as precursors for aroma compound formation [19].

## 4. Conclusions

This study demonstrated that the implementation of a traditional FFP from eastern Lanna on the mature tea leaves, an abandoned waste from tea production in western Lanna, is successful. The selected strains of *A. niger* MLF3, *L. pentosus* A14-6, and *C. rhodanensis* P3 play an important role in the biotransformation of mature tea leaves into more value-added bioactive and beneficial compounds via the capability in synthesizing and secretion of polysaccharide-degrading enzymes, β-mannanase, cellulase, pectinase, and xylanase, which are responsible for breaking down the complex structure of mature tea leaves into monosaccharides and led to increased reducing sugars during both solid state fermentation and co-culture in submerged fermentation. Additionally, *A. niger* MLF3 and *C. rhodanensis* P3 generated high levels of gallic acid, total polyphenols, total tannins, and antioxidant activity. In contrast, *L. pentosus* A14-6 was specifically involved in lactic acid production during co-culture in the later step of fermentation. The evaluation of taste and aroma by E-tongue and E-nose also confirmed the influences of the selected microorganisms, resulting in significant improvements in flavor, reduced bitterness, and enhanced aroma under controlled conditions. Integrating controlled fermentation with single- and mixed-culture inoculation provides a systematic framework for regulating the conversion of primary metabolites into diverse volatiles and for generating differentiated aroma profiles, with potential health benefits for fermented tea products. Moreover, knowledge of the biochemical changes that occur during fermentation, which may affect nutrient composition and, consequently, the properties of the final product, will serve as a valuable database for the functional foods and pharmaceutical industries. Further metabolomic analyses are essentially needed to clarify these changes and their impact on product quality and functionality.

## Figures and Tables

**Table 1:** Organic acid contents of mature tea leaves during co-fermentation at 30 °C for 7 days.

Samples	Organic Acid Content (g/L)			
	Oxalic Acid	Glucuronic Acid	Lactic Acid	Gallic Acid
C	1.01 ± 0.08 ^d^	0.33 ± 0.01 ^ab^	0	0.53 ± 0.09 ^c^
L	0.87 ± 0.09 ^d^	0.18 ± 0.03 ^b^	0.89 ± 0.01 ^b^	0
Y	1.48 ± 0.07 ^cd^	0.51 ± 0.01 ^a^	1.60 ± 0.03 ^a^	6.54 ± 0.08 ^a^
F	2.18 ± 0.01 ^ab^	0.21 ± 0.04 ^b^	0	2.70 ± 0.06 ^b^
FL	1.73 ± 0.04 ^bc^	0.49 ± 0.08 ^a^	1.89 ± 0.02 ^a^	0.12 ± 0.05 ^c^
FY	2.40 ± 0.03 ^a^	0.47 ± 0.08 ^a^	1.17 ± 0.07 ^ab^	5.25 ± 0.05 ^a^
FLY	1.02 ± 0.02 ^d^	0.34 ± 0.09 ^ab^	0.98 ± 0.01 ^b^	0.04 ± 0.09 ^c^

Note: The contents of organic acids are presented as the mean value ± standard deviation. Different superscript letters are statistically different at *p* < 0.05.

## Data Availability

The original contributions presented in this study are included in the article/Supplementary Materials. Further inquiries can be directed to the corresponding authors.

## Supplementary Materials

The following supporting information can be downloaded at: https://www.mdpi.com/article/10.3390/foods15091562/s1, Figure S1: The HPLC chromatograms for organic acid analysis (UV 210 nm). Chromatogram of the mixed organic acid standards (acetic, citric, gallic, glucuronic, lactic, malic, oxalic, succinic, and tartaric acids) and internal standard (barbituric acid) (A). Chromatogram of a representative fermentation broth sample (FY, Day 7) (B); Table S1: Key possible aroma compounds identified from seven fermentation broth samples.
