# Supplementary material for: Valorization and Functional Enhancement of Mature Assam Tea Leaves Through Indigenous Filamentous Fungi-Based Fermentation for Functional Drink Development
Source: Foods. 2026 May 1;15(9):1562. doi: 10.3390/foods15091562 (PMC13163280; doi:10.3390/foods15091562)
Supplement: Supplementary file 1 [file foods-15-01562-s001.zip › foods-4239075-supplementary.pdf]

Supplementary Material

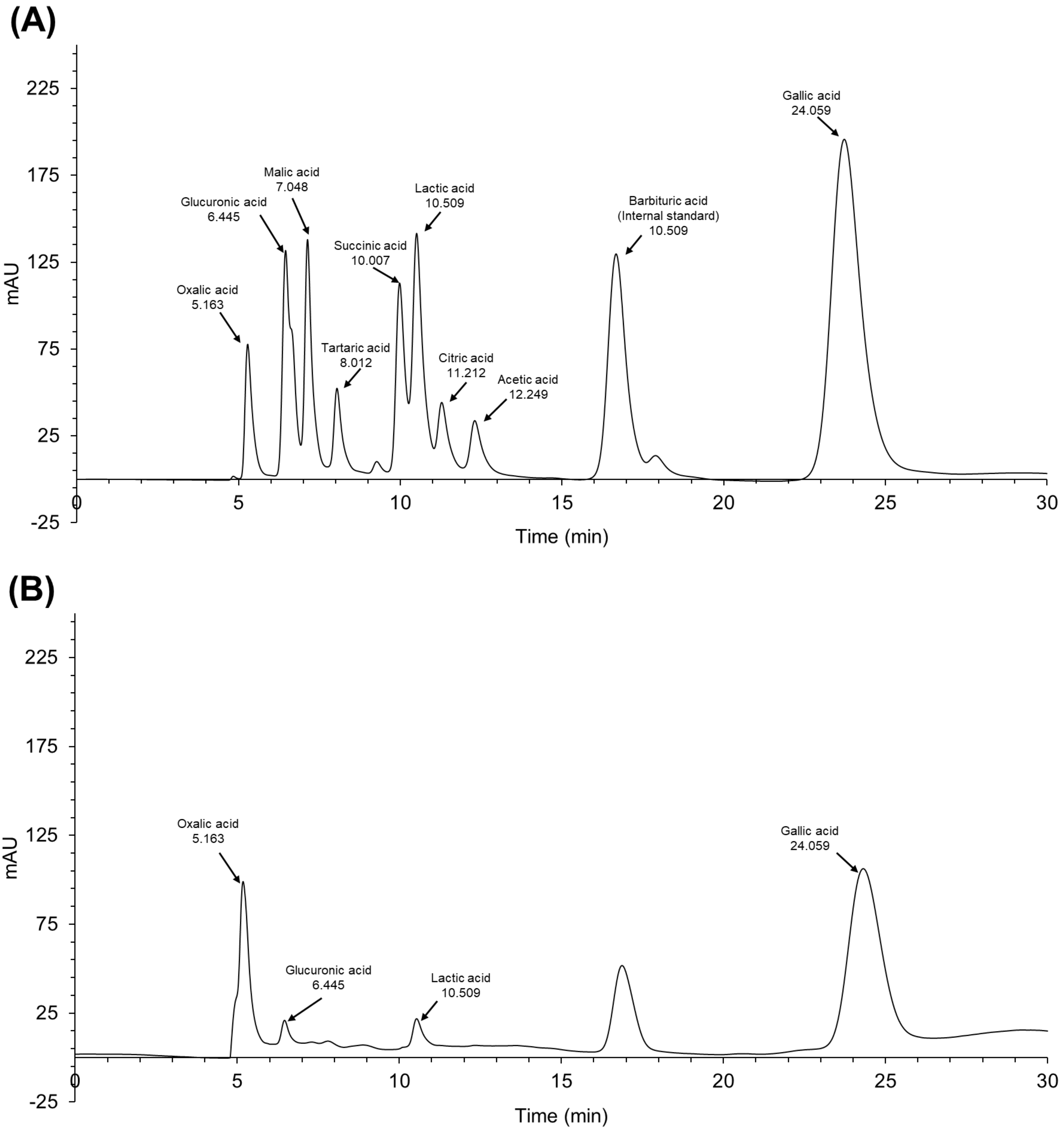

Figure S1. The HPLC chromatograms for organic acid analysis (UV 210 nm). Chromatogram of the mixed organic acid standards (acetic, citric, gallic, glucuronic, lactic, malic, oxalic, succinic, and tartaric acids) and internal standard (barbituric acid) (A). Chromatogram of a representative fermentation broth sample (FY, Day 7) (B).

Table S1. Key possible aroma compounds identified from seven fermentation broth samples

No.	Possible Compounds	Oder description	RT (s)	Average peak area						
				Control	L	Y	F	FL	FY	FLY
1	Methanol	Alcoholic, Pungent, Strong	15.94	nd	602.36	745.4	2329.24	nd	2197.49	nd
2	Ethanol	Alcoholic, Ethanol, Etheral	17.27	1549.55	5904.05	596.93	108328.7	nd	88627.63	10665.28
3	Propan-2-one	Apple, Characteristic, Fruity,	18.41	1927	nd	nd	nd	nd	nd	nd
4	Pentane	Alkane, Gasoline, Pleasant	18.49	nd	nd	nd	nd	nd	2081.28	nd
5	Ethanethiol	Earthy, Fruity, Garlic	19.84	nd	nd	691.82	nd	nd	nd	nd
6	Methyl acetate	Blackcurrant, Etheral, Fragrant	19.85	nd	nd	nd	33255.91	nd	29377.34	25778.63
7	2-methylpropanal	Almond, Apple, Burnt	20.01	nd	nd	nd	nd	24493.81	nd	nd
8	1-propanol	Alcoholic, Ethanol, Fermented	20.93	nd	nd	nd	nd	nd	802.04	nd
9	Butane-2,3-dione	Butter, Caramelized, Chlorine	22.91	nd	1112.53	nd	nd	nd	nd	nd
10	Butan-2-one	Acetone, Butter, Cheese	23.58	nd	nd	647.69	nd	nd	nd	nd
11	Acetic acid	Acetic, Acidic, Odorless,	23.84	nd	nd	nd	nd	504.8	nd	nd
12	Ethyl acetate	Acidic, Butter, Caramelized	24.78	nd	nd	1564.2	180679.85	16551.46	122874.38	12435.27
13	Methyl propanoate	Apple, Etheral, Fresh, Fruity	24.81	nd	1245.76	nd	nd	nd	nd	nd
14	Hexane	Alkane, Etheral, Gasoline	25.32	nd	nd	618.27	nd	nd	nd	nd
15	1-Propanol, 2-methyl-	Alcoholic, Bitter, Chemical	25.90	nd	nd	nd	9836.59	nd	7752.67	nd
16	Methyl isobutyrate	Apple, Floral, Fruity, Pineapple	31.30	nd	nd	nd	1631.27	nd	1692.58	nd
17	Heptane	Alkane, Fruity, Gasoline	32.25	nd	nd	543.01	nd	nd	nd	nd
18	Propyl acetate	Apple, Banana, Ester	34.14	nd	nd	nd	1006.7	nd	1154.61	nd
19	Acetoin	Butter, Coffee, Creamy	34.27	nd	664.59	nd	nd	nd	nd	nd
20	S(-)2-methyl-1-butanol	Fresh, Fruity, Malty	37.12	nd	nd	nd	18320.22	1004.5	nd	792.81
21	Ethyl isobutyrate	Apple, Floral, Fruity	39.63	nd	nd	nd	6399.63	nd	4637.29	nd
22	2,3-Butanediol	Acrid, Almond, Berry, Fermented	42.81	nd	nd	nd	3603.82	nd	nd	nd
23	Butanoic acid	Cucumber, Fruity, Green	44.25	nd	nd	nd	751.56	nd	544.55	nd
24	Ethyl isovalerate	Anise, Apple, Blackcurrant	49.51	nd	nd	nd	1542.88	nd	nd	nd
25	(Z)-2-Hexen-1-ol	Caramelized, Fruity, Green	50.01	nd	nd	nd	nd	nd	1944.08	nd

Table S1. *Cont.*

No.	Compounds	Oder description	RT (s)	Average peak area						
				Control	L	Y	F	FL	FY	FLY
26	Isoamyl acetate	Apple, Banana, Ester, Fresh	51.94	nd	nd	nd	7696.77	719.63	5336.3	581.01
27	2-butylfuran	Fruity, Mild, Spicy, Sweet	53.35	nd	nd	nd	nd	nd	969.9	nd
28	Cyclohexanone	Acetone, Minty, Peppermint	53.37	nd	nd	2694.88	1268.55	572.84	nd	nd
29	Heptan-2-ol	Acrid, Arctic bramble, Cheese	54.16	nd	1078.68	1783.22	3064.83	1349.55	1646.55	811.49
30	5-methylfurfural	Acidic, Almond, Burnt sugar	60.85	501.9	nd	nd	nd	nd	756.09	nd
31	Beta-Pinene	Dry, Fragrant, Green	60.88	nd	nd	nd	847.8	775.45	nd	646.77
32	Decane	Alkane, Fruity, Fusel, Sweet	61.59	nd	nd	1525.66	nd	nd	nd	nd
33	Terpinolene	Citrus, Fresh, Fruity	67.92	nd	nd	nd	nd	nd	507.68	nd
34	2-nonanol	Cheese, Citrus, Creamy	69.10	nd	1361.72	nd	nd	1666.06	nd	1974.09
35	Linalool	Anise, Bergamot, Citrus	69.13	nd	nd	3229.54	2077.85	nd	2174.44	nd
36	(Z)-3-Hexenyl isobutyrate	Apple, Etheral, Fruity, Green	71.19	688.43	nd	nd	nd	nd	nd	nd
37	Anisyl alcohol	Alkane, Fusel, Gasoline	79.17	nd	nd	nd	879.1	nd	nd	nd
38	[E]-whiskey lactone	Dry, Fragrant, Green	79.71	nd	nd	nd	736.39	nd	nd	nd
39	Cinnamaldehyde	Cinnamon, Clove, Pungent	80.21	nd	nd	nd	1001.92	nd	508.65	nd
40	Anethole	Anise, Herbaceous, Licorice	80.22	nd	nd	nd	nd	nd	nd	510.03
41	2-undecenal	Anise, Apple, Blackcurrant	82.23	nd	nd	nd	1096.49	nd	nd	nd
42	Tetradecane	Alkane, Fusel, Sweet	84.41	nd	nd	nd	503.63	nd	nd	nd
43	Beta-caryophyllene	Fruity, Green, Musty	88.52	nd	nd	nd	576.34	nd	nd	nd
44	Delta-Undecalactone	Alcoholic, Etheral, Medicinal	93.23	nd	nd	nd	1232.28	nd	nd	nd

nd = not detected.
